# Circulating metabolites in progression to islet autoimmunity and type 1 diabetes

**DOI:** 10.1007/s00125-019-04980-0

**Published:** 2019-08-23

**Authors:** Santosh Lamichhane, Esko Kemppainen, Kajetan Trošt, Heli Siljander, Heikki Hyöty, Jorma Ilonen, Jorma Toppari, Riitta Veijola, Tuulia Hyötyläinen, Mikael Knip, Matej Orešič

**Affiliations:** 1 Turku Bioscience, University of Turku and Åbo Akademi University, Tykistokatu 6, FI-20520 Turku, Finland; 2 Steno Diabetes Center Copenhagen, Gentofte, Denmark; 3 Children’s Hospital, University of Helsinki and Helsinki University Hospital, Stenbäckinkatu 11, 00029 HUS, Helsinki, Finland; 4 Research Program Unit, University of Helsinki, Helsinki, Finland; 5 Faculty of Medicine and Life Sciences, University of Tampere, Tampere, Finland; 6 Fimlab Laboratories, Pirkanmaa Hospital District, Tampere, Finland; 7 Immunogenetics Laboratory, Institute of Biomedicine, University of Turku, Turku, Finland; 8 Clinical Microbiology, Turku University Hospital, Turku, Finland; 9 Institute of Biomedicine, Centre for Integrative Physiology and Pharmacology, University of Turku, Turku, Finland; 10 Department of Pediatrics, Turku University Hospital, Turku, Finland; 11 Department of Pediatrics, PEDEGO Research Unit, Medical Research Centre, University of Oulu, Oulu, Finland; 12 Department of Children and Adolescents, Oulu University Hospital, Oulu, Finland; 13 Department of Women’s and Children’s Health, Karolinska Institutet, Stockholm, Sweden; 14 Department of Chemistry, Örebro University, Örebro, Sweden; 15 Tampere Center for Child Health Research, Tampere University Hospital, Tampere, Finland; 16 Folkhälsan Research Center, Helsinki, Finland; 17 School of Medical Sciences, Örebro University, 702 81 Örebro, Sweden

**Keywords:** Beta cell autoimmunity, Metabolomics, Type 1 diabetes

## Abstract

**Aims/hypothesis:**

Metabolic dysregulation may precede the onset of type 1 diabetes. However, these metabolic disturbances and their specific role in disease initiation remain poorly understood. In this study, we examined whether children who progress to type 1 diabetes have a circulatory polar metabolite profile distinct from that of children who later progress to islet autoimmunity but not type 1 diabetes and a matched control group.

**Methods:**

We analysed polar metabolites from 415 longitudinal plasma samples in a prospective cohort of children in three study groups: those who progressed to type 1 diabetes; those who seroconverted to one islet autoantibody but not to type 1 diabetes; and an antibody-negative control group. Metabolites were measured using two-dimensional GC high-speed time of flight MS.

**Results:**

In early infancy, progression to type 1 diabetes was associated with downregulated amino acids, sugar derivatives and fatty acids, including catabolites of microbial origin, compared with the control group. Methionine remained persistently upregulated in those progressing to type 1 diabetes compared with the control group and those who seroconverted to one islet autoantibody. The appearance of islet autoantibodies was associated with decreased glutamic and aspartic acids.

**Conclusions/interpretation:**

Our findings suggest that children who progress to type 1 diabetes have a unique metabolic profile, which is, however, altered with the appearance of islet autoantibodies. Our findings may assist with early prediction of the disease.

**Electronic supplementary material:**

The online version of this article (10.1007/s00125-019-04980-0) contains peer-reviewed but unedited supplementary material, which is available to authorised users.

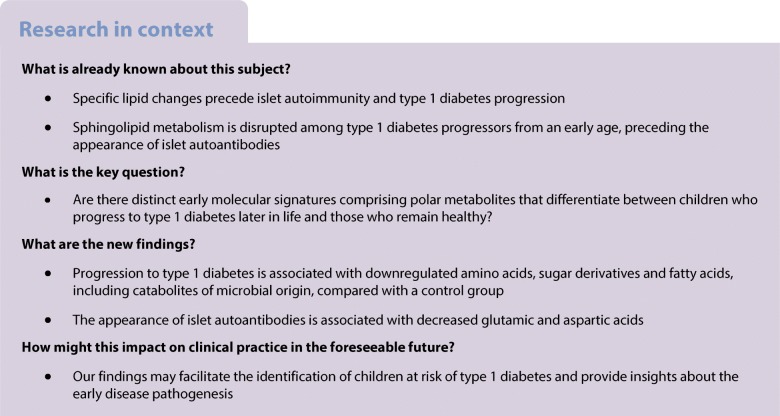



## Introduction

Type 1 diabetes is an autoimmune disease that arises as a consequence of the destruction of insulin-producing pancreatic beta cells by the immune system [1]. The incidence of type 1 diabetes is highest in children and adolescents in the developed countries [2] and an increase in disease rate is expected in young children aged under 5 years [3]. To reverse the increasing rate, early prediction and prevention of type 1 diabetes is essential. However, the aetiology of type 1 diabetes is complex and multifactorial, and the primary cause for initiation and disease progression is poorly understood [1]. Therefore, predictive and preventive measures for type 1 diabetes remain unmet medical needs.

HLA complex alleles constitute the most relevant and the strongest genetic risk factor for type 1 diabetes susceptibility [4]. However, only 3–10% of the individuals with risk HLA loci develop type 1 diabetes [5], indicating that exogenous factors such as environmental exposure, diet and gut microbiota likely play a vital role in disease progression [6]. Initiation of beta cell autoimmunity is the first detectable sign of progression towards type 1 diabetes. However, seroconversion to islet autoantibody positivity may not lead to overt diabetes [7] and the period between seroconversion and the appearance of clinical symptoms of type 1 diabetes may vary between individuals from a few months to many years [8, 9].

Previous studies suggest that children who progress to type 1 diabetes have dysregulated metabolic profiles in infancy [10–13], prior to the seroconversion for islet autoantibodies. However, studies in humans have so far mainly focused on lipids, and there is relatively little information on polar metabolites, such as those involved in central metabolic pathways, in relation to type 1 diabetes pathogenesis. Here, we study circulating polar metabolite profiles in progression to type 1 diabetes in a longitudinal study setting.

## Methods

These methods are expanded versions of descriptions in our related work [10].

### Study setting

The plasma samples were from the Finnish Type 1 Diabetes Prediction and Prevention (DIPP) study [14]. The participants in the current study were from the Tampere cohort [15]. The ethics and research committee of the participating university hospital approved the study protocol and the study followed the guidelines of the Declaration of Helsinki. Parents of all participants gave written informed consent at the beginning of the study. The samples were collected at up to six different time points, corresponding to the ages of 3, 6, 12, 18, 24 and 36 months (or above). This longitudinal cohort comprises samples from 120 children: 40 progressors to type 1 diabetes (PT1D); 40 who tested positive for at least one antibody in a minimum of two consecutive samples but did not progress to clinical type 1 diabetes during the follow-up (P1Ab); and 40 control (CTRL) children who remained islet autoantibody-negative during the follow-up until the age of 15 years. We matched the participants in the three study groups for HLA-associated diabetes risk, sex and period of birth (electronic supplementary material [ESM] Table 1). In total, we collected 415 non-fasting blood samples.

We separated plasma within 30 min of blood collection by centrifugation at 1600 *g* for 20 min at room temperature. The plasma samples were stored at −80°C until analysed.

### HLA genotyping

HLA-conferred susceptibility to type 1 diabetes was analysed using cord blood samples as described by Nejentsev et al [16]. Briefly, the HLA genotyping was performed with a time-resolved fluorometry-based assay for four alleles using lanthanide-chelate-labelled sequence-specific oligonucleotide probes detecting DQB1*02, DQB1*03:01, DQB1*03:02 and DQB1*06:02/3 alleles [17]. Carriers of DQB1*02/DQB1*03:02 or DQB1*03:02/x genotypes (here x ≠ DQB1*02, DQB1*03:01, DQB1*06:02, or DQB1*06:03 alleles) were categorised into the type 1 diabetes risk group and recruited for the follow-up programme.

### Detection of islet autoantibodies

The participants were prospectively observed for the appearance of islet cell antibodies (ICA), insulin autoantibodies (IAA), islet antigen 2 autoantibodies (IA-2A), and GAD autoantibodies (GADA), as described previously [18]. ICA were detected with the use of indirect immunofluorescence, whereas the other three autoantibodies were quantified with the use of specific radiobinding assays [19]. We used cut-off limits for positivity of 2.5 JDRF units for ICA, 3.48 relative units (RU) for IAA, 5.36 RU for GADA and 0.43 RU for IA-2A.

### Analysis of polar metabolites

After randomisation and blinding, 415 plasma (30 μl aliquot) samples were used for extraction. Polar metabolites were extracted in methanol (400 μl), as previously described [20]. For quality control and normalisation, a group-specific internal standard mix of heptadecanoic acid-d33 (175.36 mg/l), valine-d8 (35.72 mg/l), succinic acid-d4 (58.54 mg/l) and glutamic acid-d5 (110.43 mg/l) (Sigma-Aldrich, Steinheim, Germany) was added to the extraction solvent. Samples were vortexed and left to precipitate for 30 min on ice. After precipitation, extracts were centrifuged (centrifuge 5427 R; Eppendorf, Hamburg, Germany) for 3 min on 12,520 *g*. A 180 μl sample of supernatant fraction was transferred into GC vials and stored for further use. The same procedure was applied for clinic-pooled plasma, which was used for quality control and batch correction. The quantification was performed using calibration curves prepared using the following standards (Sigma-Aldrich): fumaric acid, aspartic acid, succinic acid, malic acid, methionine, tyrosine, glutamic acid, phenylalanine, arachidonic acid, isoleucine, 3-hydroxybutyric acid, glycine, threonine, leucine, proline, serine, valine, alanine, stearic acid, linoleic acid, palmitic acid and oleic acid. Standards were dissolved in methanol. The calibration curves included at least six concentration points that ranged from 1 ng/sample to 3000 ng/sample, depending on the abundance in plasma. *R*^2^ was from 97.1% up to 99.9%.

Derivatisation was performed instrumentally using an MPS2 (Gerstel, Mülheim an der Ruhr, Germany). Samples were evaporated to dryness and derivatisation was performed in two steps (details are in the ESM Methods). Derivatised compounds were analysed using a Pegasus 4D system (LECO, St Joseph, MI, USA). The method used is based on two-dimensional GC followed by high-speed time of flight acquisition of electron-ionisation-fragmented mass spectra. The primary column had internal dimensions 10 m × 0.18 mm (Rxi-5 ms, Restek Bellefonte, PA, USA) and the secondary column was 1.5 m × 0.1 mm (BPX-50, SGE Analytical Science, Austin, TX, USA). The system was guarded by a retention gap column of deactivated silica (internal dimensions 1.7 m, 0.53 mm, fused silica deactivated; Agilent Technologies, CA, USA). The modulator used nitrogen gas, which was cryogenically cooled. The second dimension cycle was 4 s. The temperature programme started at 50°C (2 min), then a gradient of 7°C up to 240°C was applied and then finally a gradient of 25°/min to 300°C, where it was held stable for 3 min. The temperature programme of the secondary column was maintained at 20°C higher than the primary column. The acquisition rate was kept at 100 Hz. The instrument was guided by ChromaTOF software (version 4.32, LECO), which was also used for calculating the area under the peaks with SN>100 and the identification of potential peaks using National Institute of Standards and Technology 14 mass spectral library and in-house library. The processing method included calculation of retention indices. Selected compounds were quantified against external calibration curves, after normalisation with internal standards, and the rest of the metabolites were normalised against internal standards, as described by Hartonen et al [20].

Results were exported as text files for further processing with Guineu [21] software; we used a 70 cut-off for peak detection and the missing values were imputed using the nearest-neighbour method in MATLAB 2017b (Mathworks, Natick, MA, USA), using default variable in the statistical toolbox. A total of 75 pooled human plasma samples were analysed for quality control purposes after every tenth sample. In addition, blank samples were analysed after every sixth sample and standard samples were analysed in each batch. The relative SD (RSD) of the concentrations was <30% for all quantified metabolites in the quality control samples, and the raw variation of the internal standards in all of the samples was also <30%. The overall %CVs across the analysis (all 415 samples) are shown in ESM Table 2.

### Data analysis

All statistical analyses were performed on log-transformed data. The transformed data were mean centred and auto scaled prior to multivariate analysis. The multivariate analysis was done using the PLS Toolbox 8.2.1 (Eigenvector Research, Manson, WA, USA) in MATLAB 2017b. ANOVA-simultaneous component analysis (ASCA) was performed to induce different factors such age, sex, case and their interaction [22]. ASCA is a multivariate extension of ANOVA used for univariate data analysis. The ASCA method exploits different factors in the experimental design, for example, sex and age, to allow easy interpretation of the variation induced by these factors and their interactions in multivariate datasets [22].

A Wilcoxon rank-sum test was performed for comparing the two study groups of samples (e.g. PT1D vs P1Ab) in a specific age cohort. For comparison, one sample per participant, closest to the age within the time window, has been used in each test. Paired Student’s *t* test was performed for the matched groups of samples (e.g. before vs after seroconversion). The resulting nominal *p* values were corrected for multiple comparisons using the Benjamin and Hochberg approach [23]. Adjusted *p* values <0.1 (*q* values) were considered significantly different among the group of hypotheses tested in a specific age cohort. All of the univariate statistical analyses were computed in MATLAB 2017b using the statistical toolbox. The fold difference was calculated by dividing the mean concentration of a metabolite species in one group by another: for instance, mean concentration in the PT1D group by the mean concentration in the P1Ab group, and then illustrated by heat maps. The locally weighted regression plot was made using smoothing interpolation function loess (with span = 1) available from the ggplot2 [24] package in R [25]. The individual metabolite levels were visualised as a box plot using GraphPad Prism 7 (GraphPad Software, La Jolla, CA, USA).

Pathway analysis of the significant metabolites (nominal *p* values <0.05) was performed in MetaboAnalyst 4.0 [26]. The compounds unmatched during compound name matching were excluded from the subsequent pathway analysis. We implemented globaltest hypergeometric testing for the functional enrichment analysis. The pathway topological analysis was based on the relative betweenness measures of a metabolite in a given metabolic network and for calculating the pathway impact score. Based on the impact values from the pathway topology analysis, the impact value threshold was set to >0.10.

## Results

### Impact of age on circulating metabolome

**Fig. 1:**
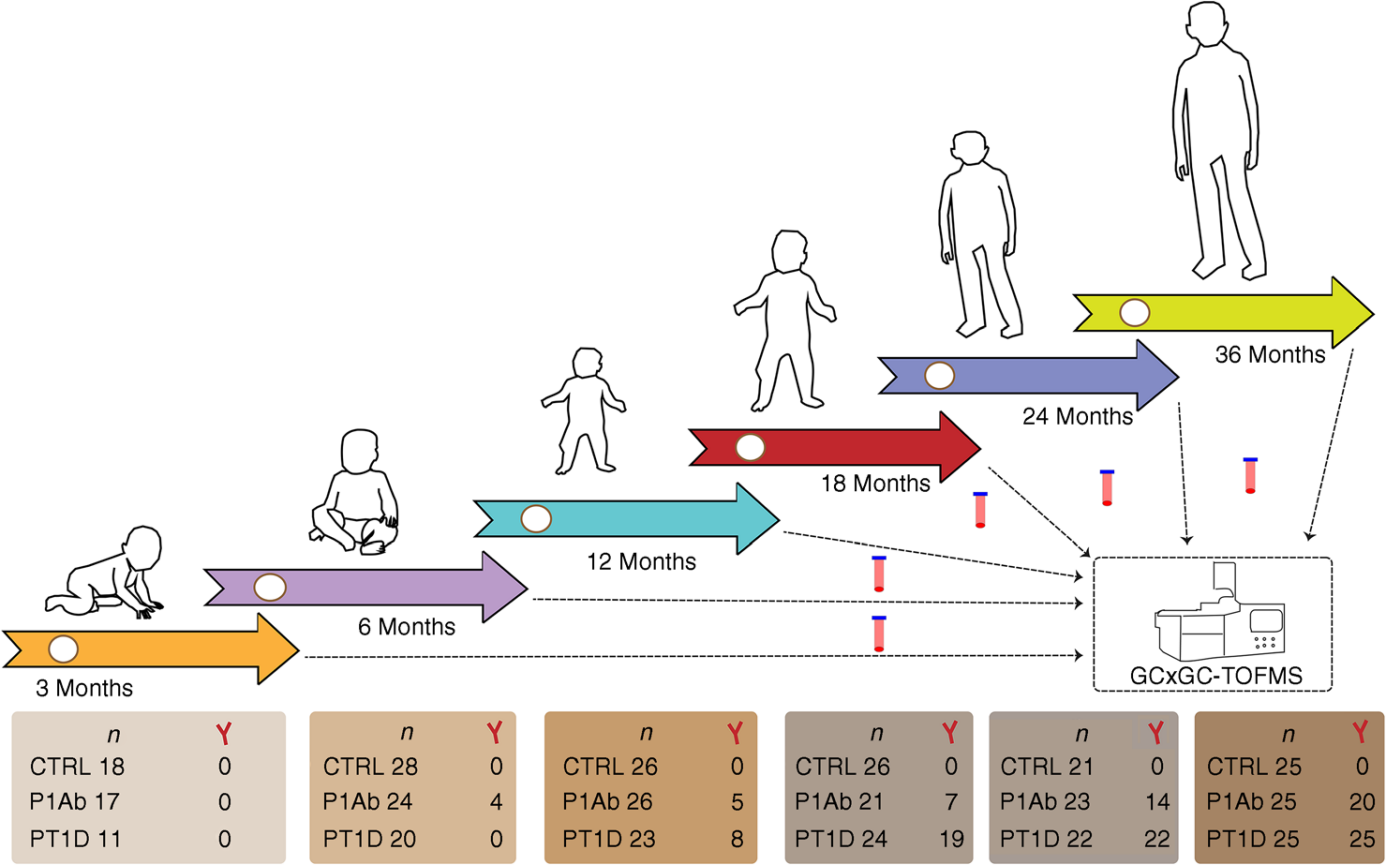
An overview of the study design. The study cohort comprised children who progressed to type 1 diabetes, children who seroconverted to one islet autoantibody but did not progress to type 1 diabetes during the follow-up, and a group who remained islet-autoantibody-negative during the follow-up until the age of 15 years. For each child, longitudinal plasma samples were drawn, corresponding to the ages of 3, 6, 12, 18, 24 and 36 months. In each age cohort and study group, the number of autoantibody-positive children is marked and represented with a Y (antibody) shape. TOF, time of flight

We performed a metabolomics analysis of polar metabolites in plasma from 120 children, divided into three study groups: those who progressed to type 1 diabetes (*n* = 40); those who seroconverted to at least one autoantibody positivity but without clinical symptoms of type 1 diabetes (*n* = 40); and a matched antibody-negative control group (*n* = 40). For each participant, plasma samples were collected corresponding to the ages of 3, 6, 12, 18, 24 and 36 months (Fig. 1). We detected metabolites (*n* = 94) from across a wide range of chemical classes, including amino acids, carboxylic acids (mainly NEFA and other organic acids), hydroxyacids, phenolic compounds, alcohols and sugar derivatives.

Principal component analysis (PCA) [27] of the complete dataset including identified metabolites displayed an age-dependent pattern (ESM Fig. 1). To resolve the impact of age on the plasma metabolome, we performed ASCA by incorporating three factors—age, sex and study group (CTRL, P1Ab, PT1D)—and their interactions. As expected, age-related variation displayed the maximum effect (4.2%, nominal *p* = 0.001) in the circulating metabolome compared with the impact of the other two factors, study group (1.2%, nominal *p* = 0.001) and sex (0.5%, nominal *p* = 0.002). Notably, the interaction factor ‘age and group’ also showed a significant effect (2.9%, nominal *p* = 0.033), while interactions between other factors (age/sex and group/sex) remained insignificant (nominal *p* values: *p* = 0.508 and *p* = 0.221, respectively).

**Fig. 2:**
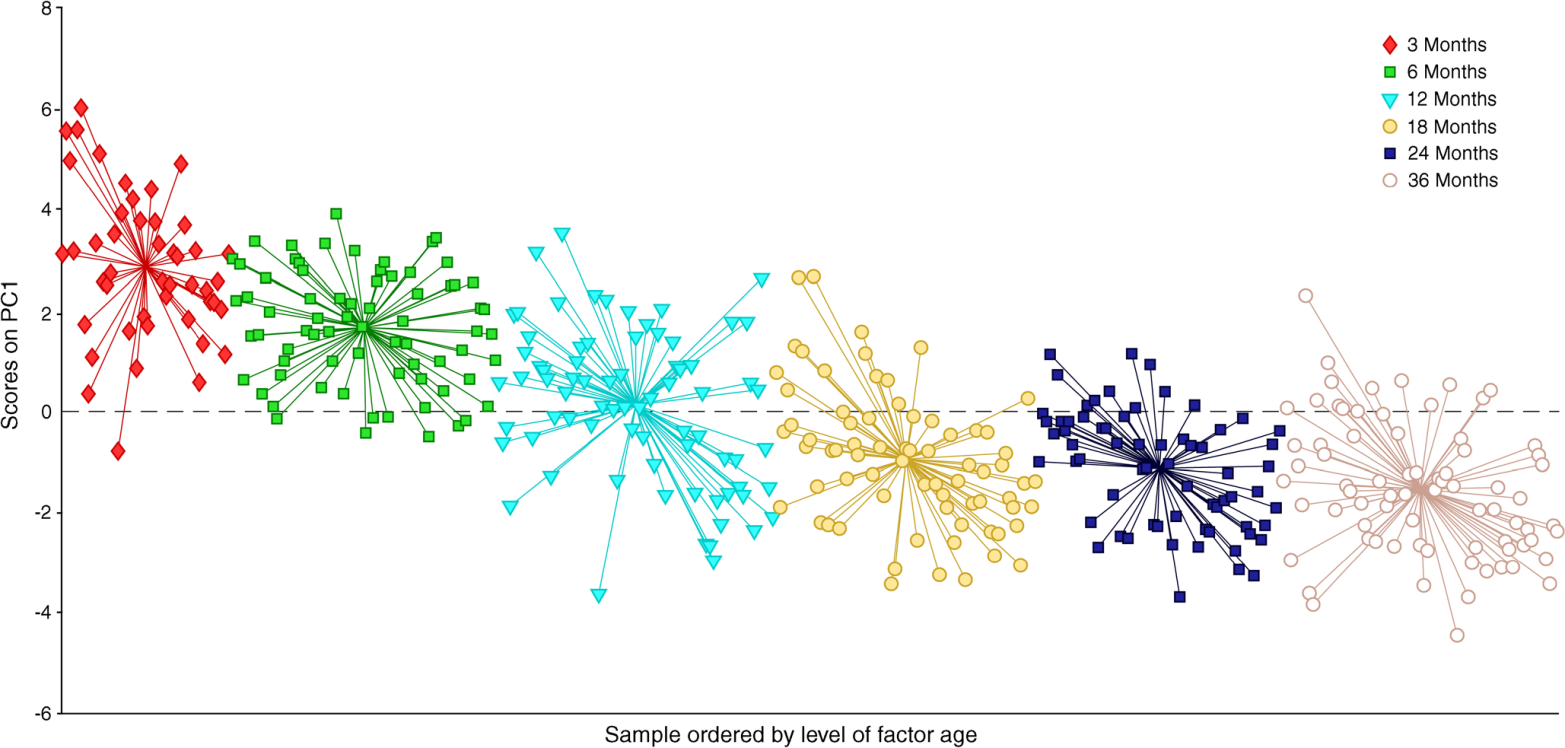
PCA score plots of the factor age, based on ASCA. These scores represent the metabolomics dataset arranged according to age in the PCA score plot. Each sample is represented by a point and coloured according to the age. The *x*-axis shows the samples arranged by factor age while the *y*-axis represents the sample score. Samples with similar scores cluster together

The scores from the first principal component (PC1) of the factor ‘age’ clearly showed an age-related trajectory in the circulatory metabolites (Fig. 2). The loading revealed high levels of branched-chain amino acids (BCAA) in the 18, 24 and 36 month age cohorts, whereas tryptophan, 3-indoleacetic acid (tryptophan derivative) and carboxylic acids (mainly NEFA) were elevated during early infancy (at 3 and 6 months). However, we did not detect any age-dependent patterns in phenolic compounds, alcohols, hydroxyacids or sugar derivatives (ESM Fig. 2).

### Metabolite profiles during progression to islet autoimmunity and type 1 diabetes

**Fig. 3:**
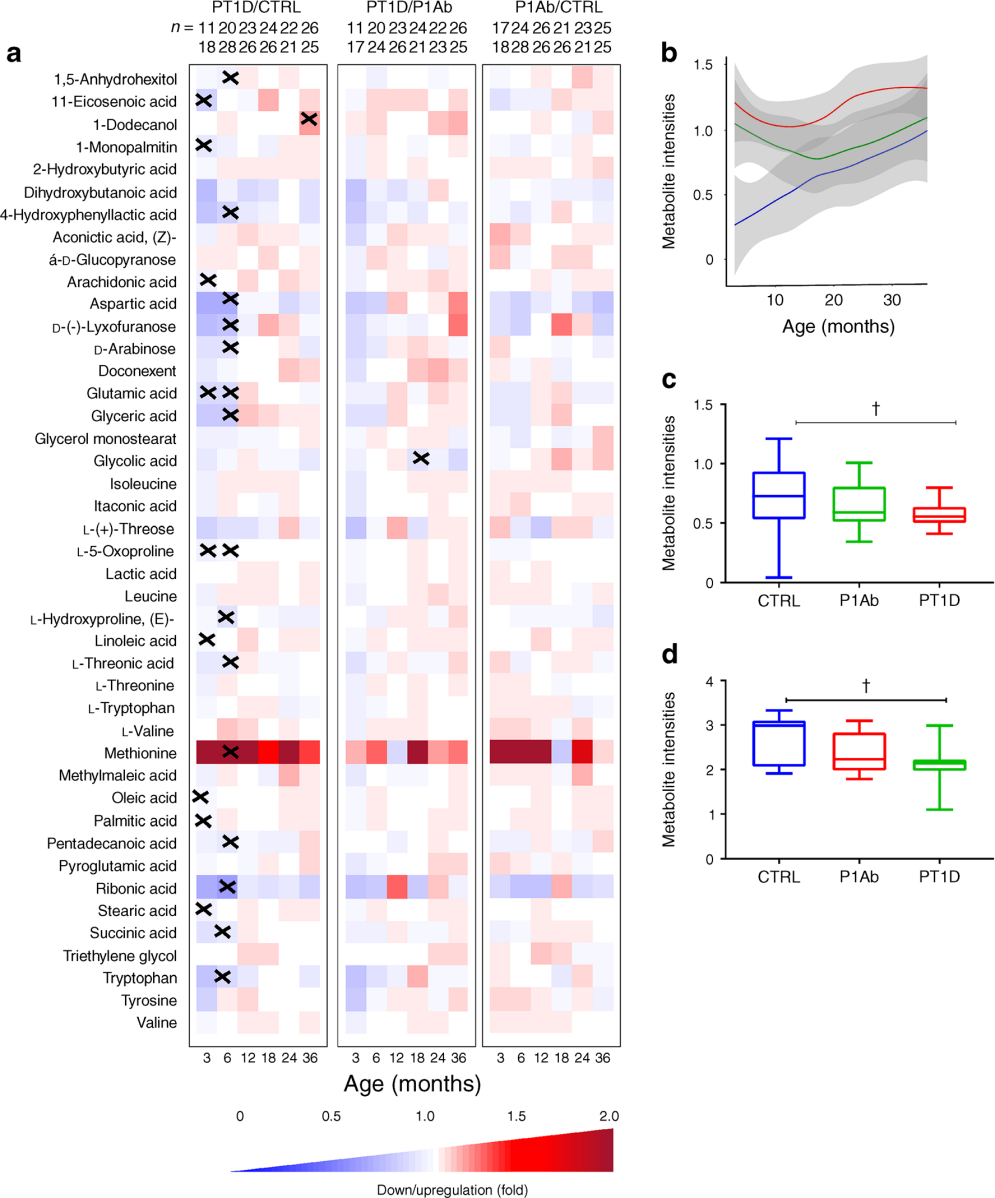
Comparison of metabolomes in three study groups in different age cohorts. (a) Heat map showing 43 metabolites representative of different metabolic classes that change between PT1D, P1Ab and CTRL. Differences in metabolite concentrations were calculated by dividing the mean concentration of a metabolite species in one group by another (PT1D/CTRL, PT1D/P1Ab or P1Ab/CTRL). The *n* values on the top row represent the numerators and *n* values on the bottom row are the denominators for each calculation. Crosses indicate adjusted *p* < 0.1. (b) Local polynomial regression fitting (LOESS) curve plot of methionine concentration over time for the three study groups. Blue, CTRL; green, P1Ab; red, PT1D. Solid line, mean value; grey shaded area, 95% CI. (c) Concentration of 4-hydroxyphenyllactic acid at 6 months of age. (d) Concentration of glutamic acid at 6 months of age. In (c) and (d), the line within each box represents the median, and the top and bottom of the box represent the 75th and 25th percentiles, respectively. The whiskers indicate the maximum and minimum values. ^†^Adjusted *p* < 0.1

Considering age as a major confounder in the plasma metabolome, we performed age-matched comparisons between the three study groups (CTRL, P1Ab and PT1D). Univariate analysis revealed that all major metabolite classes, including amino acids, NEFA and sugar derivatives, were altered in children who later progressed to type 1 diabetes, with these changes evident in infancy (Fig. 3). Altogether, 15 metabolites were different between PT1D and CTRL groups at 3 months of age (nominal *p* value <0.05). Nine out of 15 metabolites were significantly lower at 3 months in the PT1D compared with the CTRL group (adjusted *p* < 0.1; false discovery rate [FDR] threshold of 0.1) (Fig. 3, ESM Table 3). In order to assess if sex had an impact on plasma metabolite levels in children at 3 months of age, we carried out an ASCA with, as factors, study group and sex, and their interaction. When evaluating the statistics from these factors, we found only study group had a significant effect (nominal *p* = 0.012), while sex and its interaction remained insignificant (nominal *p* values: *p* = 0.081 and *p* = 0.73, respectively). The score of the factor ‘study group’ showed distinct metabolic clusters between PT1D, P1Ab and CTRL, suggesting that specific metabolic changes precede islet autoimmunity and type 1 diabetes. The loadings disclosed that methionine, 2-ketoisocaproic acid, bisphenol A (BPA), pyruvic acid, glycerol-2-phosphate and levoglucosan were higher in the PT1D group compared with the P1Ab and CTRL groups (ESM Fig. 3).

At 6 months of age, altogether 20 metabolites differed between PT1D and CTRL (nominal *p* value <0.05). Fifteen of these circulating metabolites passed the FDR threshold of 0.1 (Fig. 3a–d, ESM Table 4), including several amino acids, sugar derivatives, NEFA and various other organic acids. The levels of most of these metabolites decreased in type 1 diabetes progressors during the same period compared with CTRL. Only methionine was found to be increased in PT1D compared with CTRL at the age of 6 months (Fig. 3b). In addition, multivariate ASCA revealed that only study group (CTRL, P1Ab and PT1D) had a significant effect (nominal *p* = 0.004) on the plasma metabolites of children aged 6 months, whereas the impact of sex (nominal *p* = 0.180) and its interaction with study group (nominal *p* = 0.269) remained insignificant.

Next, we sought to examine whether children across the three study groups had altered plasma metabolite levels in the age cohorts of 12, 18, 24 and 36 months. With the exceptions of 1-dodecanol and glycolic acid, no other statistically significant differences between the study groups were observed (adjusted *p* < 0.1; FDR threshold of 0.1). At 36 months of age, the dodecanol level was higher in the PT1D compared with the CTRL group. Meanwhile, glycolic acid was lower in the PT1D compared with the P1Ab group at 18 months of age. However, these metabolites showed inconsistent trends in the longitudinal series (Fig. 3).

**Fig. 4:**
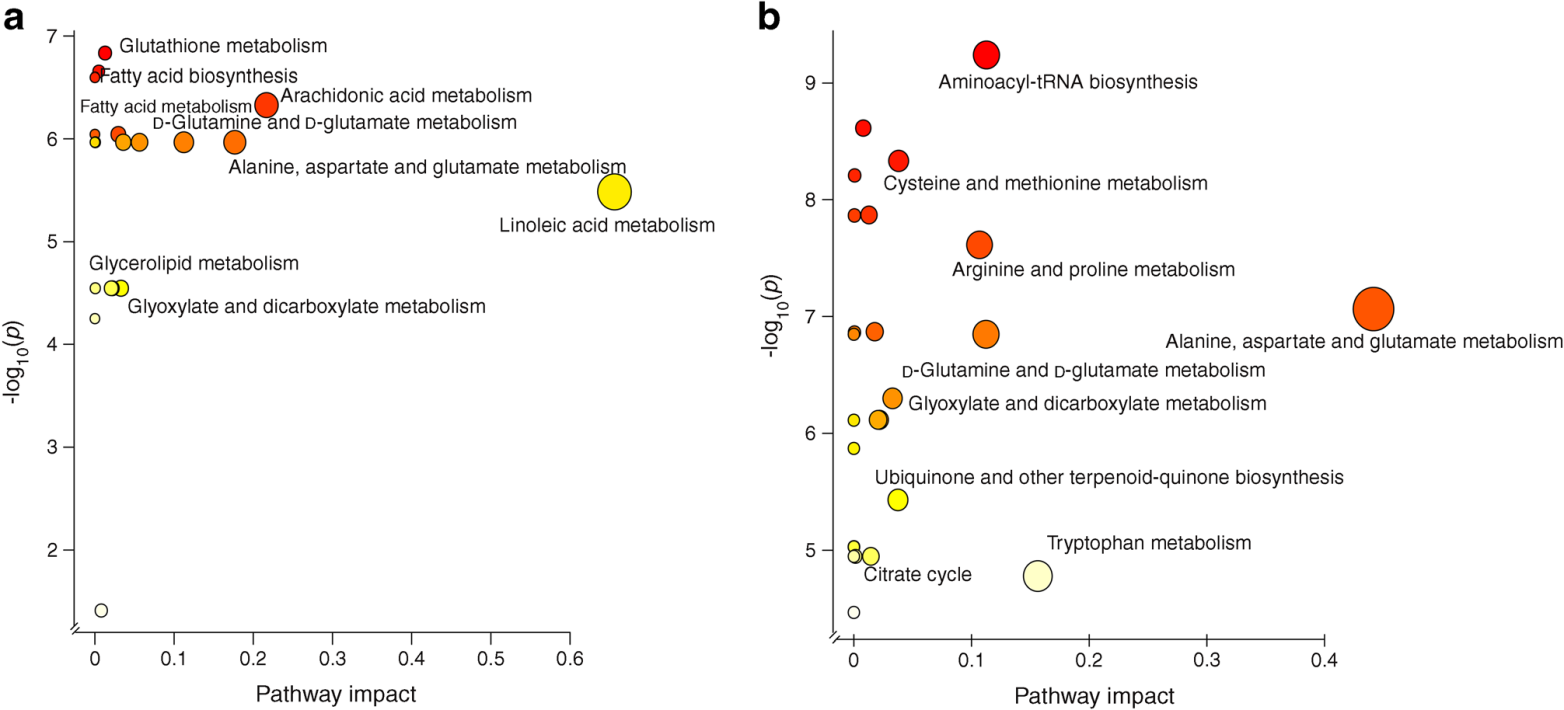
Pathway analysis of metabolites for which there were significant differences in levels between the CTRL and PT1D groups at (a) 3 and (b) 6 months of age (nominal *p* < 0.05). The pathways are shown according to the *p* values from the pathway enrichment analysis and pathway impact values from the pathway topology analysis. The metabolic pathways with impact value >0.1 were considered the most relevant pathways involved. Pathway impact values were calculated from pathway topology analysis using MetaboAnalyst. Circle size represents the pathway impact (the larger the circle, the higher the impact) and the colour gradient, from red to yellow, indicates the –log_10_(*p* value), with deep red indicating the highest –log_10_(*p* value) and pale yellow indicating the lowest. tRNA, transfer RNA

We also studied whether the pattern of a group of metabolites seen at an early age was associated with a specific metabolic pathway. The altered metabolites (nominal *p* < 0.05) between CTRL and PT1D at 3 and 6 months of age were subjected to metabolic pathway analysis (MetPA) in MetaboAnalyst [26]. In line with findings at the individual metabolite level, we found that four metabolic pathways remained altered between PT1D and CTRL groups at the age of 3 months (Fig. 4a, ESM Table 5): linoleic acid metabolism; arachidonic acid metabolism; alanine, aspartate and glutamate metabolism; and d-glutamine and d-glutamate metabolism Similarly, at 6 months of age, MetPA revealed that alanine, aspartate and glutamate metabolism, d-glutamine and d-glutamate metabolism, tryptophan metabolism, arginine and proline metabolism, as well as aminoacyl-tRNA biosynthesis remained dysregulated between the CTRL and PT1D groups (Fig. 4b, ESM Table 6).

### Metabolome before and after the first appearance of islet autoantibodies

**Fig. 5:**
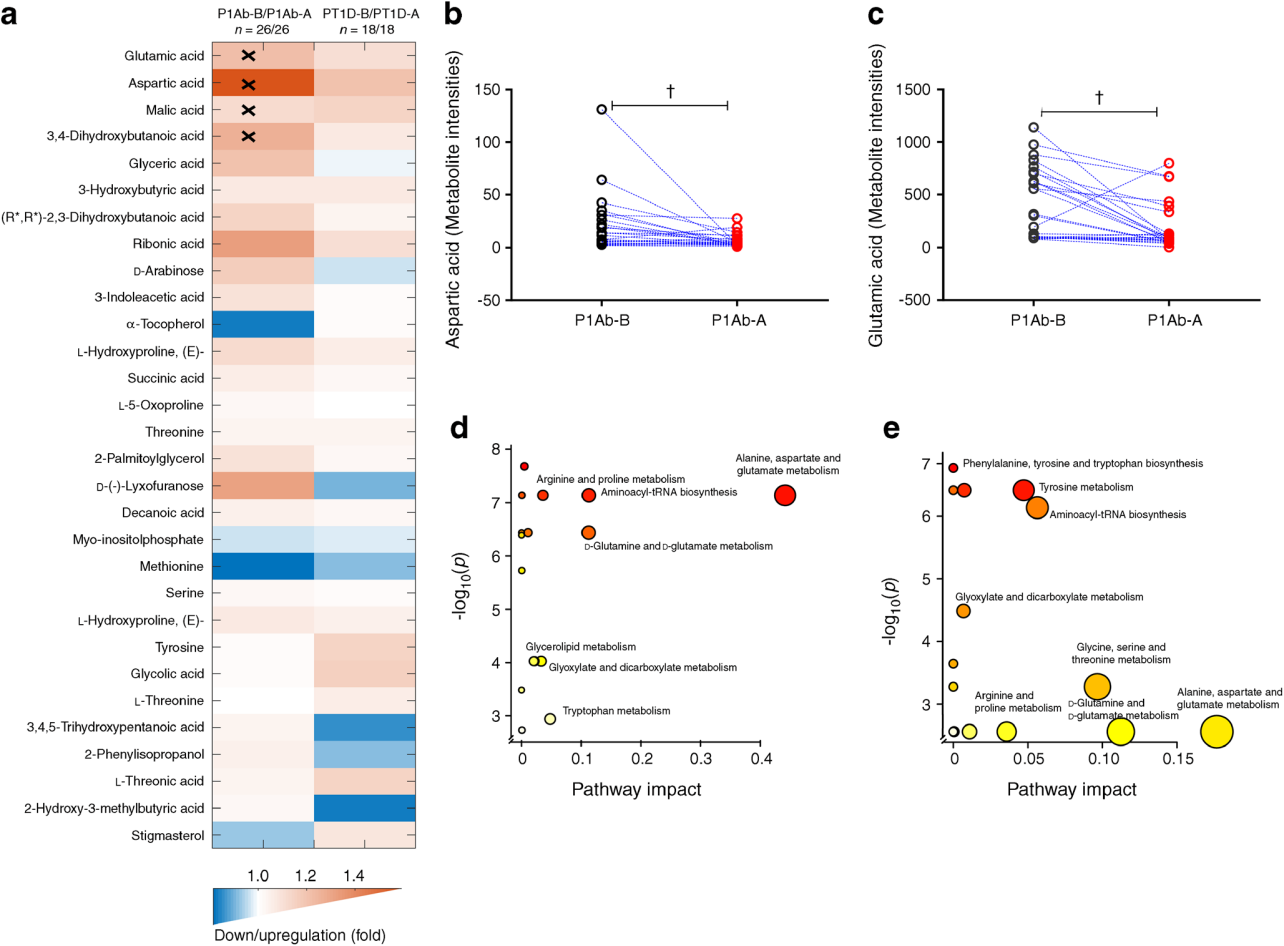
The effect of islet autoantibody positivity on metabolite profiles. (a) The most discriminating metabolites between the last available samples obtained before the first islet autoantibody appeared and the first available samples after the emergence of the first islet autoantibody in the P1Ab and PT1D groups. Crosses indicate adjusted *p* < 0.1. The pairwise scatter plot of (b) aspartic and (c) glutamic acid before and after the first appearance of islet autoantibodies. Pathway enrichment analysis of differentially expressed metabolites before and after seroconversion in (d) P1Ab and (e) PT1D. The pathways showing the largest difference include alanine, aspartate and glutamate metabolism. Circle size represents the pathway impact (the larger the circle, the higher the impact) and the colour gradient, from red to yellow, indicates the –log_10_(*p* value), with deep red indicating the highest –log_10_(*p* value) and pale yellow indicating the lowest. ^†^Adjusted *p* < 0.1-A, after seroconversion; B, before seroconversion, tRNA, transfer RNA

In order to study the effect of islet seroconversion on the metabolome, we compared metabolite levels before and after the appearance of first islet autoantibody in P1Ab and PT1D groups. Pairwise comparison revealed that 11 metabolites were altered by seroconversion in the P1Ab group (nominal *p* value <0.05, ESM Table 7), with four passing the FDR threshold of 0.1 (glutamic, aspartic, malic and 3,4-dihydroxybutanoic acids) (Fig. 5a–c). We detected seven metabolites altered before and after islet autoantibody appearance in the PT1D group (nominal *p* value <0.05), but none of these passed the FDR threshold of 0.1 (ESM Table 8). Metabolic pathway analysis corroborated these findings and revealed that alanine, aspartate and glutamate metabolism were altered when comparing the pathways before and after seroconversion within P1Ab and PT1D groups (Fig. 5d, e). However, the level of impact for these pathways varied between the P1Ab and PT1D groups, with impact values 0.441 and 0.176, respectively. Other relevant pathways and their impact are summarised in ESM Tables 9 and 10. When examining metabolite level changes in relation to the appearance of specific islet autoantibodies (ICA, IAA, islet antigen 2 autoantibodies [IA-2A] and GADA), no specific associations were identified, which may be a consequence of the small number of cases per individual autoantibody.

## Discussion

Our study identified specific metabolic disturbances in children who progressed to type 1 diabetes compared with an age-matched control group and children who developed a single islet autoantibody but did not progress to type 1 diabetes during follow-up. We found that such metabolic dysregulation exists before the first signs of islet autoimmunity. In agreement with earlier studies [10, 28, 29], a strong association was observed between the metabolome and age. We identified a distinct plasma amino acid profile in PT1D children, particularly at the ages of 3 and 6 months. Glutamic and aspartic acids as well as tryptophan remained downregulated during early infancy in the PT1D group compared with the CTRL group, but not the P1Ab group. In our previous study of polar metabolites in type 1 diabetes progression, we found no significant difference in different age cohorts when comparing PT1D and CTRL groups [13]; this may, however, be attributable to the small number of individuals in the metabolomics part of that study. Notably, and in agreement with the previous study, we also observed that the appearance of islet cell autoantibodies was associated with the downregulation of aspartic and glutamic acids [13], also corroborated by observed change in alanine, aspartate and glutamate metabolism in the MetPA.

Our findings are consistent with a previous study suggesting that amino acid dysregulation precedes the appearance of islet autoantibodies and progression to type 1 diabetes [12]. Several NEFA were also downregulated at 3 months of age. During basal metabolic processes, triacylglycerols are broken down to fatty acids and glycerol [30]. Fatty acids act as an important fuel source for cells, which is required to maintain systematic energy homeostasis [31]. Usually, under conditions when carbohydrate availability is limited, the fatty acids are an alternative substrate for energy production [32]. Here, the decrease in fatty acids may be an indication of increased energy demand in individuals in the PT1D group, further substantiated by the diminishment of circulating sugar derivatives as well as altered linoleic acid metabolism and arachidonic acid metabolism. This is also in line with our previous report [10] associating downregulated triacylglycerols and phospholipids in the PT1D group, supporting the view that altered energy metabolism is involved in the initiation of the autoimmune process and type 1 diabetes.

Accumulating evidence suggests that perturbations in the gut microbial structure are associated with, and contribute to, the pathogenesis of beta cell autoimmunity and overt type 1 diabetes [33–35]. Here, we found that 4-hydroxyphenyllactic acid [36, 37], 11-eicosenoic acid [38] and succinic acid [39], metabolites of potential microbial origin (catabolites), were significantly downregulated at an early age (3 and 6 months) in the PT1D group (nominal *p* < 0.05). The tryptophan-derived microbial catabolite 3-indoleacetic also appeared to be downregulated in PT1D (nominal *p* value <0.05, ESM Fig. 4). As catabolites generated by the gut microbes are vital to the intestinal homeostasis [37, 40], dysregulated microbial catabolism may contribute to the dysbiosis associated with progression to type 1 diabetes.

While most of the amino acids were downregulated in the PT1D group compared with the CTRL and P1Ab groups, methionine remained persistently upregulated in type 1 diabetes progressors. This appears to be in disagreement with previous studies in BABYDIAB and Environmental Triggers for Type 1 Diabetes (MIDIA) cohorts, which showed a decreased level of methionine in autoantibody-positive individuals and type 1 diabetes progressors, respectively [29, 41]. This discrepancy may, however, be explained: [1] the BABYDIAB study compared children seroconverting early in life (≤2 years) with those who developed autoantibodies at an older age; while [2] the MIDIA study highlighted differences that were mainly linked to the age of the children and the duration of breastfeeding [41]. We performed a similar comparison to that of BABYDIAB in the current study setting but found no significant differences between the groups (nominal *p* > 0.05).

The observed differences suggest disrupted methionine metabolism in the PT1D group. Methionine can be salvaged endogenously by protein/homocysteine degradation, polyamine synthesis or by the transsulfuration pathway [42], and the disturbances in these pathways could modulate neonatal epigenetic processes, including the DNA methylation and chromatin remodelling and consequently influence various immunological responses [43].

Multivariate ASCA revealed that plasma BPA was upregulated in the PT1D group, though univariate analysis across different age cohorts did not reveal significant changes (nominal *p* > 0.05) between the groups. Studies in an experimental model of autoimmune diabetes suggest that increased BPA exposure is associated with accelerated development of autoimmune diabetes [44, 45]. However, we consider that, at the present stage, our findings on the association of BPA and type 1 diabetes are inconclusive, because: [1] in our study setting we could not control for the effect of sample storage on the plasma BPA levels; and [2] the levels of BPA were not quantified. Clearly, further studies in clinical settings are merited in order to establish the effect of exposure to BPA and other environmental toxicants on the progression of type 1 diabetes or other autoimmune diseases.

A potential limitation of our study is that we could not profile microbiome or microbe-diet interactions that would be likely to influence the circulatory metabolome of the newborn infants. Future investigation of diet–microbe interactions will be needed to clarify the impact of the microbial metabolism that may potentially lower the microbial catabolites in relation to the progression of type 1 diabetes. The statistical limitations of the present study are connected with the relatively low number of identified metabolites, a relatively small sample size, which did not allow us to use a very strict FDR (<0.05) cut-off, as well as random sampling because collection of fasting samples is not feasible in infants. Nevertheless, this study generates novel hypotheses, which need further validation in larger studies within heterogeneous populations.

Taken together, while confirming several earlier findings, the present study highlights the importance of core metabolic pathways such as amino and fatty acid metabolism in the early pathogenesis of type 1 diabetes. We also observed that the appearance of islet autoantibodies does have an effect on the amino acid levels, specifically on glutamic and aspartic acids. However, these changes do not seem to be specifically associated with type 1 diabetes but are instead a general feature of islet autoimmunity, suggesting that amino acid imbalance may be a contributory factor in the initiation of autoimmunity [13]. Our study also indicates that the largest metabolic changes associated with type 1 diabetes progression have already occurred by early infancy, with these early metabolic signatures becoming less pronounced or even disappearing with age. This can be ascribed to the fact that dietary patterns could, over time, mask some of the metabolic signatures associated with type 1 diabetes or it might be related to the lack of insulin in overt type 1 diabetes. Overall, these metabolic changes are particularly apparent before the initiation of islet autoimmunity; this may have important implications for the search for early metabolic markers of type 1 diabetes and for understanding the disease pathogenesis.

## Electronic supplementary material

ESM (PDF 516 kb)

## Data Availability

The metabolomics data and the associated metadata are deposited at the MetaboLights database [46] with the acquisition number (MTBLS802). All the data supporting the findings of this study are available from the MetaboLights database or from the corresponding authors on reasonable request.

## Abbreviations

ASCA: ANOVA-simultaneous component analysis
BPA: Bisphenol A
CTRL: Control
FDR: False discovery rate
GADA: GAD autoantibodies
IAA: Insulin autoantibodies
ICA: Islet cell antibodies
MetPA: Metabolic pathway analysis
P1Ab: Individuals who tested positive for at least one antibody in a minimum of two consecutive samples but did not progress to clinical type 1 diabetes during the follow-up
PCA: Principal component analysis
PT1D: Progressors to type 1 diabetes
RU: Relative units
