# Supplementary material for: Circulating metabolites in progression to islet autoimmunity and type 1 diabetes
Source: Diabetologia. 2019 Aug 23;62(12):2287–97. doi: 10.1007/s00125-019-04980-0 (PMC6861356; doi:10.1007/s00125-019-04980-0)
Supplement: Supplementary file 1 — (PDF 516 kb) [file 125_2019_4980_MOESM1_ESM.pdf]

Circulating Metabolites in Progression to Islet Autoimmunity and Type 1 Diabetes

Electronic supplementary material

ESM Methods

Two step derivatization:

In the first step 25 µl of methoxyamine hydrochloride (TS-45950; Thermo Scientific: USA) was added to the sample. While mixing, the solution was incubated for one hour at 45 °C. In the second step, 25µl of N-methyl-N-trimethylsilyltrifluoroacetamide (Sigma-Aldrich; Steinheim, Germany) was added. Incubation was again performed for one hour at 45 °C. Before injection 50 µl of hexane was added to increase the volatility of the solvent. Additional standards here added during derivatization. n-alkanes (c = 8 mg/l in MSTFA) were used for calculation of retention indexes and 4,4′-dibromooctafluorobiphenyl (c = 9.8 mg/l in hexane) were used as syringe standard to control the quality of injection. 1 µl of derivatized sample was injected after derivatization program was completed.

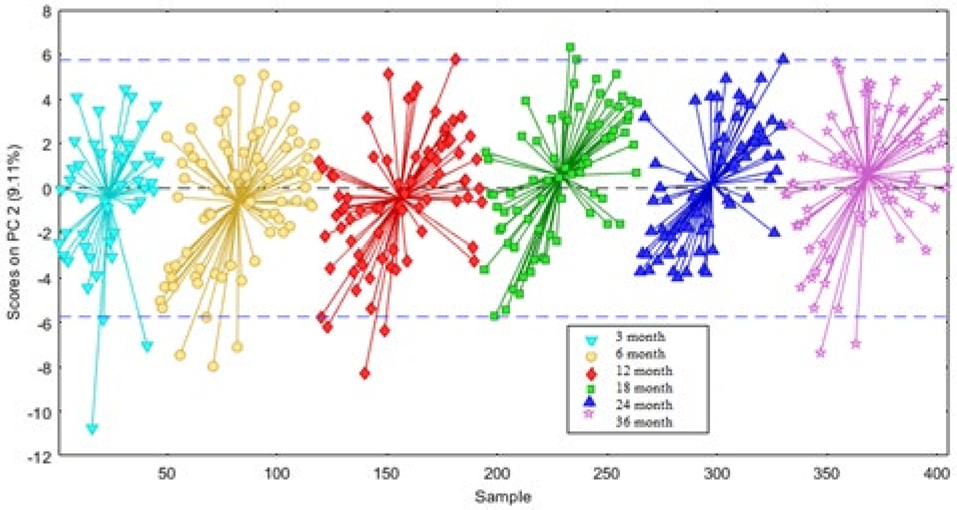

ESM Figure 1. PCA score plot of the second principal component obtained from 405 plasma samples.

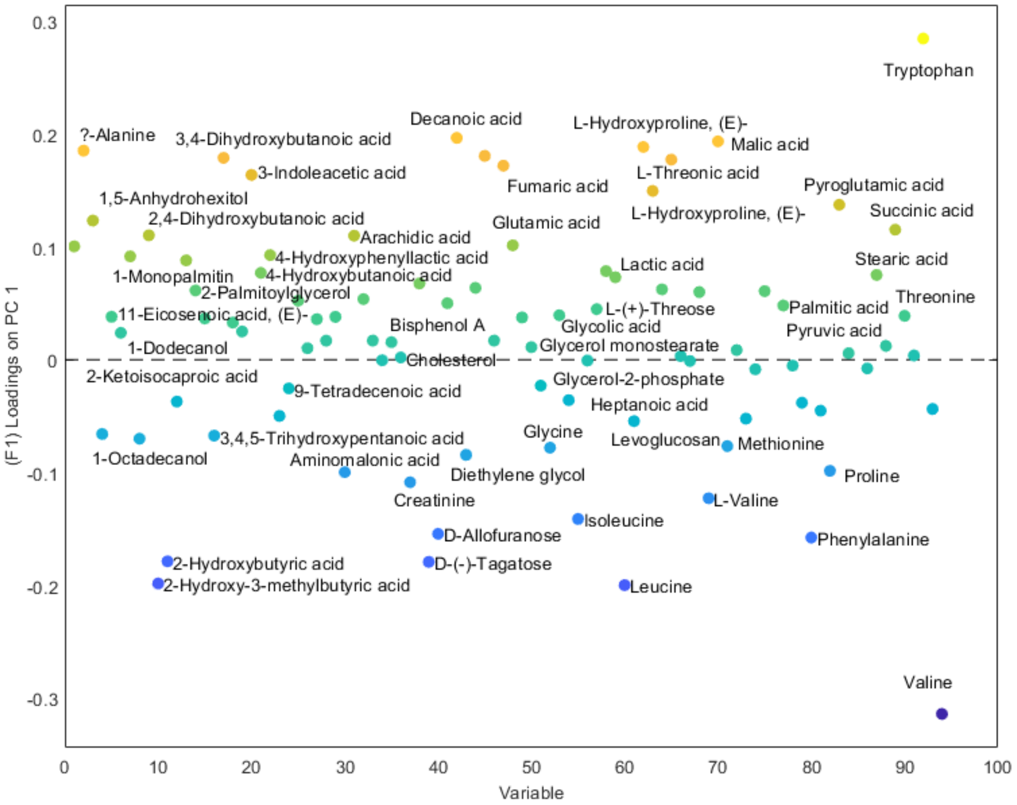

ESM Figure 2. ASCA PC1 loading plot for Fig 1. The loading explains the pattern seen in the score plot that provides the means to interpret the class specific metabolic alteration related to age. x-axis is the variable order and y-axis represents the metabolic pattern corresponding to the score plot. Here, colour of the loadings indicates the loading scores in PC1.

a

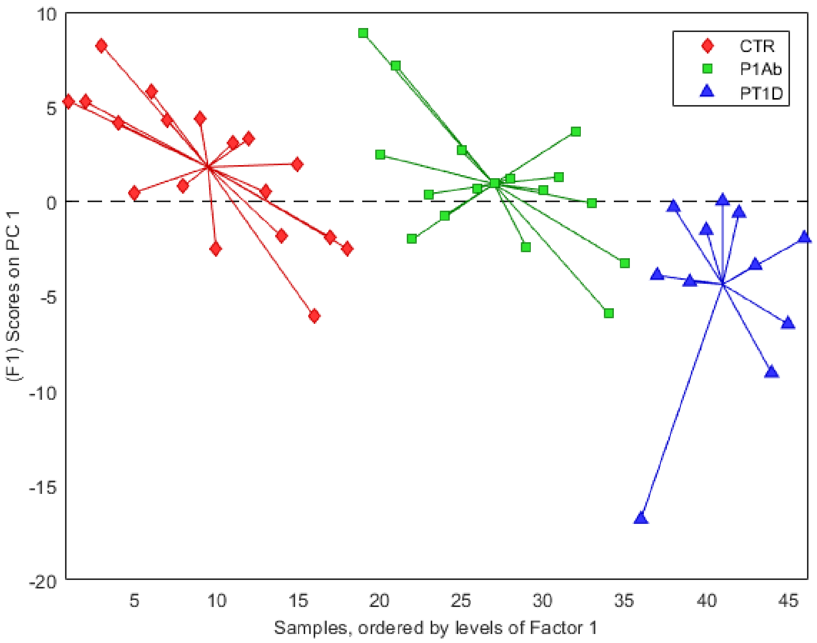

b

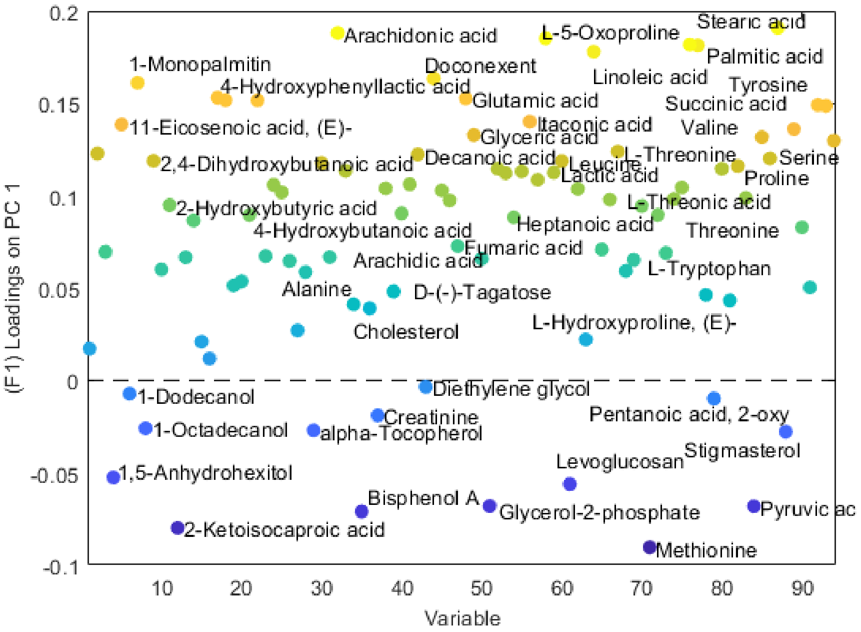

ESM Figure 3. PCA score plots and loadings based on ANOVA-simultaneous component analysis (ASCA). **(a)** PC1 score plot obtained based on the factor study cases score in ASCA analysis at age of 3 months. Here, each sample is represented by a point and coloured according to the case (red diamond: subjects who remained islet autoantibody negative during the follow-up age (CTR), green square: subjects who tested positive for at least one antibody in a minimum of two consecutive samples but did not progress to clinical T1D during the follow-up (P1Ab), blue triangle up: progressors to type-1-diabetes (PT1D). Samples with similar score are clustered together. **(b)** The corresponding PC1 loading plot. The loadings explain the pattern seen in the score plot which provides the means to interpret the metabolic alteration related to case. X-axis is the variable order and y-axis represents the metabolic pattern corresponding to the score plot. Here, colour of the loadings indicates the loading scores in PC1.

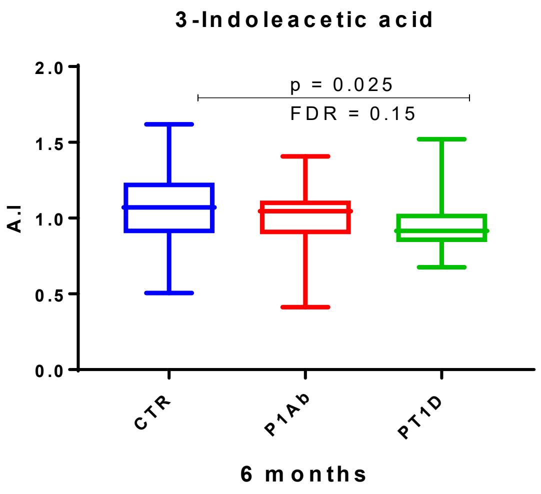

ESM Figure 4. Concentrations of 3-Indole acetic acid at 6 months of age.

ESM Table 1. Anthropometric characteristics of study population.

	**PT1D**	**P1Ab**	**CTR**
**Sex(female, male)**	(14, 26)	(14, 26)	(14, 26)
**Age at time of diagnosis (mean ± SD)**	4.75 ± 2.94	NA	NA
**Age at time of first seroconversion (mean ± SD)**	1.34 ± 0.58	3.05± 2.50	NA
**HLA information**			
**High risk (DR3 - DQA1*05 - DQB1*02) /DRB1*0401/2/4/5 - DQA1*03 - DQB1*0302)**	9	4	7
**Moderately increased risk***	21	22	14
**Slightly increased risk***	10	9	7
**Neutral***		3	4
**Decreased risk and/or not possible to form haplotype***		2	8

*other than DR3 - DQA1*05 - DQB1*02) /DRB1*0401/2/4/5 - DQA1*03 - DQB1*0302. This table has been adapted from [10]

ESM Table 2. Coefficient of variation across the analysis (415 samples).

Metabolite	% Coefficient of variation (CV)
Nonadecanoic acid	32.61
Diethylene glycol	32.7
Myo-Inositolphosphate	34.86
Fumaric acid	37.55
2-Phenylisopropanol	39.87
Valine	41.32
p-Hydroxydiisopropylbenzene	41.49
L-5-Oxoproline	43.44
á-D-(+)-Mannopyranose	44.42
Cholesterol	44.52
Serine	44.64
Aminomalonic acid	45.53
Lactic acid	45.69
Alanine	46.6
2-Palmitoylglycerol	46.89
Methylmaleic acid	47.02
3,4-Dihydroxybutanoic acid	47.68
Glycerol-2-phosphate	47.95
Itaconic acid	48.22
á-D-Glucopyranose	48.31
Octanoic acid	49.4
Threonine	49.93
Malic acid	50.06
Stearic acid	50.63
L-Threonic acid	50.88
Palmitic acid	51.35
Isoleucine	52.58
1-Dodecanol	53.48
4-Hydroxyphenyllactic acid	53.51
Linoleic acid	53.82
2-Hydroxybutyric acid	53.89
Leucine	54.34
Arachidic acid	54.44
Arachidonic acid	54.51
L-Threonine	54.52
4-Hydroxybutanoic acid	55.59
Pentadecanoic acid	56.62
Stigmasterol	57.5
Doconexent	58.25
Creatinine	59.25
1-Monopalmitin	59.73
Succinic acid	59.97

**Phenylalanine**	60.71
**Proline**	61.17
**9-Hexadecenoic acid**	61.55
**Oleic acid**	61.65
**(R*,R*)-2,3-Dihydroxybutanoic acid**	61.81
**9-Tetradecenoic acid**	61.84
**Aconitic acid, (Z)-**	63.7
**Pentanoic acid, 2-oxy**	64.1
**L-Threonic acid**	64.54
**L-Valine**	64.73
**Decanoic acid**	64.79
**D-Allofuranose**	64.9
**Glycine**	65.03
**Triethylene glycol**	65.28
**Pyroglutamic acid**	66.76
**1-Octadecanol**	67.35
**Heptanoic acid**	69.93
**2-Ketoisocaproic acid**	71.48
**D-(-)-Lyxofuranose**	72.28
**Glycerol monostearate**	72.45
**11-Eicosenoic acid, (E)-**	72.57
**L-Hydroxyproline, (E)-**	73.85
**L-Hydroxyproline, (E)-**	74.66
**3-Indoleacetic acid**	74.9
**Tyrosine**	75.16
**2,4-Dihydroxybutanoic acid**	76.21
**3,4,5-Trihydroxypentanoic acid**	77.85
**2-Oleoylglycerol**	80.69
**Glyceric acid**	81.65
**alpha-Tocopherol**	83.87
**L-(+)-Threose**	84.3
**D-Arabinose**	84.91
**1,5-Anhydrohexitol**	85.65
**L-Tryptophan**	89.45
**Dodecanoic acid**	91.4
**Tryptophan**	92.05
**3-Hydroxyisovaleric acid**	94.33
**Glutamic acid**	100.48
**3-Hydroxybutyric acid**	114.25
**Pyruvic acid**	114.32
**Methionine**	116.18
**?-Alanine**	117.19
**D-(-)-Tagatose**	120.25
**Aspartic acid**	124.85
**Azelaic acid**	127.08

**Bisphenol A**	131.44
**Levoglucosan**	133.58
**Ribonic acid**	148.97
**1,5-Anhydrohexitol**	171.48
**Ethanolamine**	194.86
**Glycolic acid**	225.34
**2-Hydroxy-3-methylbutyric acid**	245.9

Here, the median % CV (50th centile % CV) is 61.82 and the 90th centile % CV is 120.71.

ESM Table 3. Plasma metabolites different between CTR and PT1D at age 3 months.

S:N	Metabolite	Nominal p-values	Adjusted-pvalues	Log2F
1	11-Eicosenoic acid, (E)-	0.0013	0.057	-0.341
2	L-5-Oxoproline	0.0013	0.057	-0.143
3	Stearic acid	0.0021	0.057	-0.102
4	Palmitic acid	0.0024	0.057	-0.084
5	Oleic acid	0.0032	0.0616	-0.097
6	Arachidonic acid	0.0043	0.067	-0.132
7	1-Monopalmitin	0.0057	0.067	-0.186
8	Linoleic acid	0.0057	0.067	-0.095
9	Glutamic acid	0.0065	0.068	-0.312
10	3-Hydroxybutyric acid	0.023	0.22	-0.171
11	Doconexent	0.033	0.26	-0.19
12	D-Arabinose	0.037	0.26	-0.216
13	Ribonic acid	0.041	0.26	-0.755
14	L-Threonic acid	0.041	0.26	-0.162
15	Glyceric acid	0.041	0.26	-0.438

ESM Table 4. Plasma metabolites different between CTR and PT1D at age 6 months.

S:N	Metabolite	Nominal P-values	Adjusted-pvalues	Log2F
1	Ribonic acid	7.4E-05	0.0070	-0.919
2	D-Arabinose	0.00063	0.020	-0.23
3	L-Threonic acid	0.00063	0.020	-0.198
4	Methionine	0.0015	0.036	2.049
5	Pentadecanoic acid	0.0019	0.036	-0.177
6	Glyceric acid	0.0025	0.036	-0.402
7	L-5-Oxoproline	0.0027	0.036	-0.093
8	Succinic acid	0.0040	0.045	-0.187
9	1,5-Anhydrohexitol	0.00431	0.045	-0.125
10	Glutamic acid	0.0056	0.052	-0.291
11	L-Hydroxyproline, (E)-	0.0077	0.0655	-0.221
12	4-Hydroxyphenyllactic acid	0.0098	0.075	-0.334
13	Aspartic acid	0.010	0.075	-0.732
14	D-(-)-Lyxofuranose	0.014	0.093	-0.482
15	Tryptophan	0.015	0.093	-0.295
16	3-Indoleacetic acid	0.026	0.15	-0.166
17	alpha-Tocopherol	0.027	0.15	0.405
18	Malic acid	0.046	0.22	-0.085
19	9-Tetradecenoic acid	0.048	0.21	-0.223
20	Creatinine	0.048	0.26	0.153

ESM Table 5. The detailed results of potential metabolic pathways that altered between CTRL and PT1D at age 3 months

Metabolic pathway	Total Cpd	Hits	Nominal p	-log(p)	Holm adjust	FDR	Impact
Alanine, aspartate and glutamate metabolism	24	2	0.0007	7.1429	0.0166	0.002	0.441
Aminoacyl-tRNA biosynthesis	75	2	0.0007	7.14	0.016	0.002	0.112
D-Glutamine and D-glutamate metabolism	11	1	0.001	6.44	0.025	0.002	0.112
Tryptophan metabolism	79	1	0.052	2.93	0.106	0.055	0.047
Arginine and proline metabolism	77	2	0.0007	7.14	0.016	0.002	0.035
Glyoxylate and dicarboxylate metabolism	50	1	0.017	4.02	0.106	0.020	0.032
Pentose phosphate pathway	32	1	0.017	4.02	0.106	0.020	0.021
Glycerolipid metabolism	32	1	0.017	4.02	0.106	0.020	0.020
Glutathione metabolism	38	1	0.001	6.44	0.025	0.002	0.010
Butanoate metabolism	40	2	0.0004	7.67	0.0101	0.002	0.004
Ubiquinone and other terpenoid-quinone biosynthesis	36	1	0.065	2.73	0.106	0.065	0.0006
Nitrogen metabolism	39	2	0.0007	7.14	0.016	0.002	0.0006
Histidine metabolism	44	2	0.0007	7.14	0.016	0.002	0.0005
Glycine, serine and threonine metabolism	48	2	0.003	5.72	0.025	0.004	0.0004
Porphyrin and chlorophyll metabolism	104	1	0.0015	6.44	0.025	0.002	0
Cysteine and methionine metabolism	56	1	0.001	6.39	0.025	0.002	0
Lysine biosynthesis	32	1	0.001	6.39	0.025	0.002	0
beta-Alanine metabolism	28	1	0.001	6.39	0.025	0.002	0
Cyanoamino acid metabolism	16	1	0.001	6.39	0.025	0.002	0
Nicotinate and nicotinamide metabolism	44	1	0.001	6.39	0.025	0.002	0
Pantothenate and CoA biosynthesis	27	1	0.001	6.39	0.025	0.002	0
Synthesis and degradation of ketone bodies	6	1	0.030	3.48	0.106	0.033	0

ESM Table 6. The detailed results of potential metabolic pathways that altered between CTRL and PT1D at age 6 months

Metabolic pathway	Total Cpd	Hits	Nominal p	-log(p)	Holm adjust	FDR	Impact
**Alanine, aspartate and glutamate metabolism**	24	3	0.00086	7.06	0.019	0.002568	0.4416
**Tryptophan metabolism**	79	2	0.0084	4.78	0.046	0.008711	0.15633
**Arginine and proline metabolism**	77	4	0.00016	8.74	0.004	0.001581	0.11325
**Aminoacyl-tRNA biosynthesis**	75	4	9.72E-05	9.24	0.002	0.001581	0.11268
**D-Glutamine and D-glutamate metabolism**	11	1	0.0011	6.85	0.021	0.002568	0.1123
**Cysteine and methionine metabolism**	56	2	0.00024	8.33	0.006256	0.001581	0.03806
**Ubiquinone and other terpenoid-quinone biosynthesis**	36	2	0.0044	5.43	0.035311	0.005776	0.03749
**Glyoxylate and dicarboxylate metabolism**	50	2	0.0018	6.30	0.031202	0.00321	0.03291
**Pentose phosphate pathway**	32	1	0.0022	6.12	0.035311	0.00321	0.02181
**Glycerolipid metabolism**	32	1	0.0022	6.12	0.035311	0.00321	0.0206
**Butanoate metabolism**	40	2	0.0010	6.87	0.021825	0.002568	0.01774
**Citrate cycle (TCA cycle)**	20	1	0.0071	4.95	0.045828	0.007628	0.01446
**Glutathione metabolism**	38	2	0.000382	7.87	0.009171	0.00159	0.01285
**Ascorbate and aldarate metabolism**	45	2	0.00018	8.61	0.004902	0.001581	0.00802
**Propanoate metabolism**	35	1	0.0071	4.95	0.045828	0.007628	0.00134
**Nitrogen metabolism**	39	3	0.00027	8.21	0.006815	0.001581	0.00067
**Histidine metabolism**	44	2	0.0010	6.86	0.021825	0.002568	0.00051
**Glycine, serine and threonine metabolism**	48	3	0.00038	7.87	0.009171	0.00159	0.00047
**Amino sugar and nucleotide sugar metabolism**	88	1	0.0065	5.03	0.045828	0.007628	7.00E-05
**Porphyrin and chlorophyll metabolism**	104	1	0.0011	6.85	0.021825	0.002568	0
**Lysine biosynthesis**	32	1	0.0022	6.11	0.035311	0.00321	0
**beta-Alanine metabolism**	28	1	0.0022	6.11	0.035311	0.00321	0
**Cyanoamino acid metabolism**	16	1	0.0022	6.11	0.035311	0.00321	0
**Nicotinate and nicotinamide metabolism**	44	1	0.0022	6.11	0.035311	0.00321	0
**Pantothenate and CoA biosynthesis**	27	1	0.0022	6.11	0.035311	0.00321	0
**Tyrosine metabolism**	76	2	0.0028	5.87	0.035311	0.003895	0
**Pentose and glucuronate interconversions**	53	1	0.0065	5.03	0.045828	0.007628	0
**Phenylalanine metabolism**	45	1	0.0071	4.95	0.045828	0.007628	0
**Phenylalanine, tyrosine and tryptophan biosynthesis**	27	1	0.011	4.47	0.045828	0.011493	0

ESM Table 7. Plasma metabolites different between B-P1Ab and A-P1Ab.

S:N	Metabolite	Nomincal p-values	Adjusted-p-values
1	Glutamic acid	0.00017	0.016
2	Aspartic acid	0.0015	0.047
3	Malic acid	0.0015	0.047
4	3,4-Dihydroxybutanoic acid	0.0022	0.052
5	Glyceric acid	0.010	0.19
6	3-Hydroxybutyric acid	0.016	0.26
7	(R*,R*)-2,3-Dihydroxybutanoic acid	0.024	0.29
8	Ribonic acid	0.025	0.29
9	D-Arabinose	0.040	0.38
10	3-Indoleacetic acid	0.048	0.38
11	alpha-Tocopherol	0.049	0.38

Abbreviations: Before seroconversion in P1Ab (B-P1Ab), after seroconversion in P1Ab (A-P1Ab).

ESM Table 8. Plasma metabolites different between B-PT1D and A-PT1D.

S:N	Metabolite	Nominal-p-values	Adjusted p-values
1	Malic acid	0.0017	0.16
2	Tyrosine	0.0037	0.17
3	Glycolic acid	0.0074	0.19
4	L-Threonine	0.0080	0.19
5	3,4,5-Trihydroxypentanoic acid	0.023	0.40
6	Glutamic acid	0.026	0.40
7	2-Phenylisopropanol	0.044	0.60

Abbreviations: Before seroconversion in progressors (B-PT1D), after seroconversion in progressors (A-PT1D).

ESM Table 9. The detailed results of potential metabolic pathways that altered between B-P1Ab and A-P1Ab.

Metabolic pathway	Total Cpd	Hits	Nominal p	-log(p)	Holm adjust	FDR	Impact
**Alanine, aspartate and glutamate metabolism**	24	2	0.00079	7.1429	0.0166	0.002455	0.4416
**Aminoacyl-tRNA biosynthesis**	75	2	0.00079	7.1429	0.0166	0.002455	0.11268
**D-Glutamine and D-glutamate metabolism**	11	1	0.001595	6.4409	0.025519	0.002455	0.1123
**Tryptophan metabolism**	79	1	0.052983	2.9378	0.10694	0.055506	0.0478
**Arginine and proline metabolism**	77	2	0.00079	7.1429	0.0166	0.002455	0.03582
**Glyoxylate and dicarboxylate metabolism**	50	1	0.017823	4.0273	0.10694	0.020637	0.03291
**Pentose phosphate pathway**	32	1	0.017823	4.0273	0.10694	0.020637	0.02181
**Glycerolipid metabolism**	32	1	0.017823	4.0273	0.10694	0.020637	0.0206
**Glutathione metabolism**	38	1	0.001595	6.4409	0.025519	0.002455	0.01095
**Butanoate metabolism**	40	2	0.000462	7.6796	0.010168	0.002455	0.0048
**Ubiquinone and other terpenoid-quinone biosynthesis**	36	1	0.065137	2.7313	0.10694	0.065137	0.00069
**Nitrogen metabolism**	39	2	0.00079	7.1429	0.0166	0.002455	0.00067
**Histidine metabolism**	44	2	0.00079	7.1429	0.0166	0.002455	0.00051
**Glycine, serine and threonine metabolism**	48	2	0.003253	5.7281	0.025519	0.004473	0.00047
**Porphyrin and chlorophyll metabolism**	104	1	0.001595	6.4409	0.025519	0.002455	0
**Cysteine and methionine metabolism**	56	1	0.001674	6.3928	0.025519	0.002455	0
**Lysine biosynthesis**	32	1	0.001674	6.3928	0.025519	0.002455	0
**beta-Alanine metabolism**	28	1	0.001674	6.3928	0.025519	0.002455	0
**Cyanoamino acid metabolism**	16	1	0.001674	6.3928	0.025519	0.002455	0
**Nicotinate and nicotinamide metabolism**	44	1	0.001674	6.3928	0.025519	0.002455	0
**Pantothenate and CoA biosynthesis**	27	1	0.001674	6.3928	0.025519	0.002455	0
**Synthesis and degradation of ketone bodies**	6	1	0.030629	3.4858	0.10694	0.033692	0

ESM Table 10. The detailed results of potential metabolic pathways that altered between B-PT1D and A-PT1D.

Metabolic pathway	Total Cpd	Hits	Nominal p	-log(p)	Holm adjust	FDR	Impact
**Alanine, aspartate and glutamate metabolism**	24	1	0.077339	2.5596	0.46403	0.077339	0.17664
**D-Glutamine and D-glutamate metabolism**	11	1	0.077339	2.5596	0.46403	0.077339	0.1123
**Glycine, serine and threonine metabolism**	48	1	0.037713	3.2778	0.3017	0.058283	0.09661
**Aminoacyl-tRNA biosynthesis**	75	3	0.00216	6.1376	0.026103	0.005246	0.05634
**Tyrosine metabolism**	76	1	0.001631	6.4183	0.026103	0.004622	0.04724
**Arginine and proline metabolism**	77	1	0.077339	2.5596	0.46403	0.077339	0.03582
**Glutathione metabolism**	38	1	0.077339	2.5596	0.46403	0.077339	0.01095
**Phenylalanine, tyrosine and tryptophan biosynthesis**	27	1	0.001631	6.4183	0.026103	0.004622	0.00738
**Glyoxylate and dicarboxylate metabolism**	50	1	0.011235	4.4887	0.11235	0.023874	0.00686
**Histidine metabolism**	44	1	0.077339	2.5596	0.46403	0.077339	0.00051
**Nitrogen metabolism**	39	2	0.001151	6.7676	0.019558	0.004622	0
**Ubiquinone and other terpenoid-quinone biosynthesis**	36	1	0.001631	6.4183	0.026103	0.004622	0
**Phenylalanine metabolism**	45	1	0.001631	6.4183	0.026103	0.004622	0
**Thiamine metabolism**	24	1	0.001631	6.4183	0.026103	0.004622	0
**Porphyrin and chlorophyll metabolism**	104	2	0.026077	3.6467	0.23469	0.049257	0
**Valine, leucine and isoleucine biosynthesis**	27	1	0.037713	3.2778	0.3017	0.058283	0
**Butanoate metabolism**	40	1	0.077339	2.5596	0.46403	0.077339	0
